# Evaluating the reliability and validity of the 12-item WHODAS 2.0 among people with mental health conditions in seven low- and middle-income countries: analysis of secondary data

**DOI:** 10.1192/bjo.2025.10778

**Published:** 2025-10-01

**Authors:** Awoke Mihretu, Sarah Aleyan, Jessica Schmider, Charlotte Hanlon, Crick Lund, Ricardo Araya, Alicia White, Kassahun Habtamu

**Affiliations:** Department of Psychiatry, School of Medicine, College of Health Sciences, Addis Ababa University, Ethiopia; Economist Impact, The Economist Group, Dubai, United Arab Emirates; Economist Impact, The Economist Group, London, UK; Division of Psychiatry, Centre for Clinical Brain Sciences, University of Edinburgh, UK; Centre for Innovative Drug Development and Therapeutic Trials for Africa (CDT-Africa), College of Health Sciences, Addis Ababa University, Ethiopia; Centre for Global Mental Health, Health Service and Population Research Department, Institute of Psychiatry, Psychology and Neuroscience, King’s College London, UK; Alan J Flisher Centre for Public Mental Health, Department of Psychiatry and Mental Health, University of Cape Town, South Africa; School of Psychology, College of Education and Behavioural Studies, Addis Ababa University, Ethiopia

**Keywords:** Disability assessment, psychometric properties, low- and middle-income countries, WHO Disability Assessment Schedule, functional impairment

## Abstract

**Background:**

The World Health Organization Disability Assessment Schedule (WHODAS 2.0) has been validated across various settings and health conditions. However, few studies have evaluated the 12-item WHODAS 2.0 within low- and middle-income countries (LMICs) among individuals with mental health conditions.

**Aims:**

This study aimed to evaluate the psychometric properties of the 12-item WHODAS 2.0 in populations with depression, anxiety and psychosis from seven LMICs.

**Method:**

Secondary analyses were carried out using existing longitudinal data-sets in adult populations with depression, anxiety and psychosis across Brazil, Ethiopia, Ghana, India, Nigeria, Peru and South Africa. Reliability, validity and responsiveness to change of the 12-item WHODAS 2.0 were examined.

**Results:**

The 12-item WHODAS-2.0 was acceptably one-dimensional for all data-sets at baseline, with model-fit indices ranging from moderate to excellent. Internal consistency of the measure was found to be high across settings (Cronbach’s *α* = 0.83−0.97). Weak to moderate correlations with measures of symptom severity were found across all countries, except India. Moderate to strong correlations were observed with measures of functioning/quality of life across all countries, except Nigeria and Ghana.

Internal responsiveness to change was large in five out of seven studies, except both Ethiopian studies. However, external responsiveness to change exhibited variability, with weak to moderate correlations between change in WHODAS 2.0 and symptom scores across all countries.

**Conclusion:**

The 12-item WHODAS 2.0 generally showed acceptable psychometric properties across different settings and mental health conditions. However, high variability was observed in convergent validity and external responsiveness to change, which warrants further investigation.

Functional impairment is an important but often overlooked aspect of mental health conditions. It refers to the impact of an individual’s health condition on the performance of daily activities that are necessary to fulfil their roles at work, school, home or other social areas.^1^ For instance, individuals with symptoms of anxiety, depression or psychosis may experience difficulties in their social life, reduced interaction with others and challenges performing work-related activities.^2^ At the population level, depression and anxiety cause the most years lived with disability globally of any physical or mental health condition.^3^ Functioning has also been identified as an important outcome throughout the course of treatment and clinical practice and integral in enhancing perceptions of societal inclusion according to people with lived experience of mental health conditions.^4^

## Development and validation of the WHODAS 2.0

The World Health Organization Disability Assessment Schedule (WHODAS 2.0) is a measure developed to assess functional impairment across conditions, settings, and cultures.^1^ It is grounded in the International Classification of Functioning (ICF) framework and assesses an individual’s level of functioning across six life domains: (a) cognition (understanding and communicating); (b) mobility (moving and getting around); (c) self-care (paying attention to one’s hygiene, dressing, eating and staying alone); (d) getting along (interacting with other people); (e) life activities (domestic responsibilities, leisure, work and school); and (f) participation (joining in community activities and participating in society).^1^

The WHODAS 2.0 was developed and validated through an extensive cross-cultural study spanning 19 countries, in which items were selected for inclusion after exploring the contextual practicalities of health status assessment in different cultures.^5^ It has been translated into over 30 languages to enable wide use across a variety of settings. The full version of the WHODAS 2.0 contains 36 items, whereas the shortened version contains a subset of 12 items.

A systematic review evaluating the performance of the WHODAS 2.0 across different health conditions and settings provided evidence to support its reliability and validity as a measure of functional impairment.^6^ Overall, the WHODAS 2.0 showed strong alignment with other internationally recognised measures of disability and functional impairment. In light of the evidence supporting its utility, the 12-item WHODAS 2.0 was selected as a common measure for the assessment of functioning among adults with depression and anxiety by the Common Measures in Mental Health (CMMH) initiative.^7^ Despite this, there are clear gaps in the evidence in regard to the performance of the 12-item version of WHODAS 2.0 among people with mental health conditions. For instance, there is limited published evidence on the 12-item WHODAS 2.0 in several areas, including its performance in low- and middle-income countries (LMICs) and in populations with psychosis. Specifically, limited evidence exists on the concurrent, convergent and factorial validity and responsiveness to change of the 12-item version of the WHODAS 2.0 among people with mental disorders in LMICs.

It is important to understand how the WHODAS 2.0 performs in different settings as the measure was designed to be a cross-cultural measure of functioning. Other measures of functioning have garnered criticism for claiming to be universal despite being developed in high-income settings and focusing on activities that may not be relevant or applicable in low-income settings.^8^ Studies focusing on evaluating cross-cultural validity are important in establishing whether assessments of functioning are valid and reliable when used across different settings.

## Psychometric properties of the WHODAS 2.0 in people with mental health conditions

We conducted a systematic search (March 2023) across Embase, MEDLINE, PsycInfo and CINAHL to identify studies assessing the psychometric performance of the 12-item WHODAS 2.0 among populations with mental health conditions. Of the 14 primary studies identified, the majority were conducted in high-income countries (HICs),^9–18^ with only four studies conducted in LMICs.^8,19–21^ Overall, the studies identified supported the reliability and validity of the 12-item WHODAS 2.0 in their respective settings. Specifically, four studies were identified that assessed the convergent validity of the 12-item WHODAS 2.0 against measures of symptom severity in people with mental health conditions.^9,11,15,21^ Overall, the three studies from HICs suggested moderate to strong convergent validity of the WHODAS 2.0 against measures of symptom severity such as the Patient Health Questionnaire-9 (PHQ-9), while one study from India found weak correlation with the Revised Clinical Interview Schedule (CIS-R) in adults with anxiety and depression.^21^ Similarly, we found that all four studies reporting on the concurrent validity of the WHODAS 2.0 among people with mental health conditions stemmed from HICs.^9,11,14,15^ They found moderate to strong correlations between the WHODAS 2.0 and other measures of functioning and disability. A systematic review found that a one-factor structure fitted the 12-item WHODAS 2.0 in previous studies in the general population, or in groups with physical or mental health disorders,^6^ and this structure has been endorsed by the WHO.^22^

Furthermore, only one study identified in our search explicitly assessed the 12-item WHODAS 2.0’s responsiveness to change in a sample of participants with common mental disorders.^21^ This study, conducted in India, found that participants who had improvements in WHODAS 2.0 scores were more likely to have recovered from anxiety or depression based on their symptom scores, suggesting that the measure is capable of measuring changes in functional impairment associated with changes in mental health symptoms.^21^ Moving forward, additional research is needed to assess the cross-cultural validity of the 12-item WHODAS 2.0, particularly within LMICs and among populations with mental health conditions, to gain a better understanding of the applicability of this measure across different settings and health conditions.

## Rationale for conducting the study

Our study set out to fill a research gap that was identified through our evidence review along with consultations with a panel of lived-experience experts, mental health clinicians and academic experts. This was done through analysis of existing data-sets across diverse LMIC settings, with the aim of extending the current evidence on the cross-cultural validity of the WHODAS 2.0. The overall objective of this study was to evaluate the psychometric properties of the 12-item version of the WHODAS 2.0 in populations with anxiety, depression and psychosis, using secondary data from seven LMICs, including Brazil, Ethiopia, Ghana, India, Nigeria, Peru and South Africa. Specifically, this study examined the internal consistency; concurrent, convergent and factorial validity; and the internal and external responsiveness to change of the 12-item WHODAS 2.0. Based on the existing evidence base, we hypothesised that:

- The one-factor model would fit well with the 12-item WHODAS 2.0 across data sets.
- The 12-item WHODAS 2.0 would demonstrate good internal consistency.
- In regard to internal responsiveness to change, significant improvements in the 12-item WHODAS 2.0 scores would be observed over time as change is expected post-intervention.
- The 12-item WHODAS 2.0 would have moderate correlations with measures of symptom severity, functioning and quality of life.
- Changes in the 12-item WHODAS 2.0 scores over time would moderately correlate with changes in the scores of measures of symptom severity.

## Method

### Study design and setting

This study analysed existing longitudinal data drawn from seven LMICs: Brazil, Ethiopia, Ghana, India, Nigeria, Peru and South Africa.^8,23–27^ Below, we provide an overview of the data-sets included in this analysis (Table 1). Written or verbal informed consent was obtained from participants, caregivers or guardians assessed across all studies. Data-sets were selected on the basis that the study was longitudinal, assessed functioning in individuals with depression, anxiety or psychosis using either the 12- or 36-item WHODAS 2.0, and was conducted in a low- or middle-income setting.

**Table 1:** Overview of included data-sets

Study name	Language of WHODAS 2.0	Study description	Outcome assessment time points	Measures used to assess outcomes
AFFIRM validation study – Ethiopia	Amharic	A pre-post study conducted among a sample of 150 individuals with new cases of severe mental health conditions and 150 caregivers	Baseline and 6-week follow-up	Butajira Functioning Scale (BFS), Brief Psychiatric Rating Scale – Expanded version (BPRS-E), 36-item WHODAS 2.0 was administered, with only the subset of items forming the 12-item version analysed in the current study
AFFIRM (TaSCS) study – Ethiopia	Amharic	A non-inferiority randomised controlled trial (RCT) conducted in a sample of 324 individuals with severe mental health conditions (schizophrenia, schizoaffective disorder, bipolar disorder or severe major depressive disorder)	Baseline, 12--month follow-up, 18--month follow-up	BFS, BPRS-E, 12-item WHODAS 2.0
AFFIRM study – South Africa	isiXhosa	An RCT conducted in a sample of 420 women with perinatal depression from two antenatal clinics	Baseline antenatal check-up (≤28 weeks pregnant), 3 months postnatal, 12 months postnatal	Cape Town Functional Assessment Instrument for Maternal Depression, Edinburgh Postnatal Depression Scale, Hamilton Depression Rating Scale, 12-item WHODAS 2.0
CONEMO study – Brazil	Not specified	An RCT conducted in a sample of 880 individuals who had a PHQ-9 score of ≥10 and were receiving treatment for hypertension and/or diabetes at a primary care unit	Baseline, 3--month follow-up, 6-month follow-up	EuroQol-5 Dimensions (EQ-5D), Patient Health Questionnaire-9 (PHQ-9), 12-item WHODAS 2.0
CONEMO study – Peru	Not specified	An RCT in a sample of 432 individuals who had a PHQ-9 score of 10 or higher and also attended ambulatory treatment clinics for hypertension or diabetes	Baseline, 3-month follow-up, 6-month follow-up	EQ-5D, PHQ-9, 12-item WHODAS 2.0
HAP trial – India	Not specified	An RCT conducted in a sample of 495 individuals with moderately severe to severe depression	3-months follow-up, 12-months follow-up	PHQ-9, 12-item WHODAS 2.0
COSIMPO study – Nigeria and Ghana	Yoruba (Nigeria) and Akan-Twi (Ghana)	An RCT conducted in a sample of 307 (Nigeria 156, Ghana 151) individuals with psychoses	Baseline, 3-month follow-up, 6-month follow-up	Global Assessment of Functioning, Positive and Negative Syndrome Scale, 12-item WHODAS 2.0

WHODAS 2.0, World Health Organization Disability Assessment Schedule 2.0; AFFIRM, Africa Focus on Intervention Research for Mental Health; TaSCS Task Sharing for the Care of Severe mental disorders; CONEMO, Emotional Control; HAP, Healthy Activity Program; COSIMPO, Collaborative Shared Care to Improve Psychosis.

### Measures

All of the data-sets included either the self- or interviewer-administered 12-item version of the WHODAS 2.0 (or the 36-item version from which the 12-item version could be derived). Some of the data-sets included measures of mental health symptom severity and/or other measures of functioning or quality of life. Below, we describe the specific measures utilised in each of the data-sets included.

*Mental health symptom severity measures:* The Africa Focus on Intervention Research for Mental Health (AFFIRM) Ethiopia WHODAS 2.0 validation study and the Task Sharing for the Care of Severe mental disorders in a low-income country (TaSCS) trial collected data using the Brief Psychiatric Rating Scale – Expanded version (BPRS-E) to measure a comprehensive set of clinical psychiatric symptoms of depression, anxiety and psychoses.^28^ The AFFIRM South Africa trial collected data using the 17-item Hamilton Depression Rating Scale (HDRS or HAM-D),^29^ a widely used scale for the measurement of depressive symptoms, and the Edinburgh Postnatal Depression Scale (EPDS),^30^ a screening tool for postpartum depression or anxiety. The Emotional Control (CONEMO) trial and the Health Activity Program (HAP) India trial administered the PHQ-9 to assess depression symptom severity.^31^ Lastly, the Collaborative Shared Care to Improve Psychosis (COSIMPO) trial collected data on improvements in participants’ positive and negative psychotic symptoms using the Positive and Negative Syndrome Scale (PANSS).^32^

*Functioning or quality of life measures:* The AFFIRM Ethiopia WHODAS 2.0 validation study and TaSCS trial used the Butajira Functioning Scale (BFS), a contextually appropriate and locally developed functioning scale for people with severe mental health conditions.^33^ The AFFIRM South Africa study used a locally developed functional assessment scale specific to the measurement of perinatal depression, referred to as the Cape Town Functional Assessment Instrument (CFAI) for Maternal Depression.^34^ The EuroQol-5 Dimensions (EQ-5D), an instrument evaluating an individual’s generic quality of life, was used in the CONEMO trial.^35^ Lastly, the COSIMPO trial administered the Global Assessment of Functioning (GAF) scale to participants as a mental health-specific measure of day-to-day functioning.^36^

### Statistical analysis

All statistical analyses were done using the Stata software, version 18 for Windows.

For the data-sets where item-level data was available, confirmatory factor analysis (CFA) was carried out to test whether a one-dimensional model is applicable to the 12-item WHODAS 2.0 in the data sets we included. Goodness of fit was assessed using the following indices: χ^2^-test, acceptable if χ^2^/*df* is less than 3.0; Comparative Fit Index (CFI), acceptable if its value ≥0.95; Tucker–Lewis Index (TLI), acceptable if its value exceeds 0.90; and root mean square error of approximation (RMSEA), acceptable if the value is less than 0.06. The Akaike Information Criterion (AIC) and Bayesian Information Criterion (BIC) were used to compare the model fit to the data; lower AIC or BIC values suggest a better fit relative to the model complexity.

In CFA, acceptable factor loadings vary depending on the context and field of research. Generally, a loading of ≥0.70 indicates a strong factor loading, while a loading between 0.40 and 0.70 is considered acceptable. Loadings below 0.40 are deemed weak, but slightly lower loadings (e.g. 0.30–0.40) may still indicate some relevance to the underlying factor.^37^

The internal consistency of the 12-item WHODAS 2.0 was determined using Cronbach’s *α*. Values below 0.7 were considered unacceptable, from 0.7–0.8 acceptable, 0.8–0.9 good, and above 0.9 were excellent.

To assess the convergent validity of the 12-item WHODAS 2.0 with measures of mental health symptom severity, Pearson’s correlation was used. Pearson correlation coefficient values of 0.00–0.09 indicated negligible correlation, while values of 0.10–0.39 indicated weak correlation, 0.40–0.69 moderate correlation, 0.70–0.89 strong correlation and values of 0.90–1.00 indicated a very strong correlation. To assess the concurrent validity of the 12-item WHODAS 2.0 with other measures of function or quality of life (as a proxy for function), Pearson’s correlation coefficient was computed and interpreted using the same thresholds as those used to assess convergent validity.

We evaluated both the internal and external responsiveness to change of the 12-item WHODAS 2.0 using data from the intervention arm of each randomised controlled trial (RCT) data-set. Internal responsiveness to change measures the change in WHODAS 2.0 scores over time. A paired sample *t*-test and Cohen’s *d* effect size was computed to determine the internal responsiveness to change. Cohen’s *d* scores were interpreted using the following thresholds: small (*d* ≤ 0.2), medium (*d* = 0.2–0.5), and large (*d* = 0.5–0.8). External responsiveness to change measures the correlation between changes in the WHODAS 2.0 scores and changes in the scores from related measures of symptom severity. Spearman’s rank-order correlation of the change scores over time between the 12-item WHODAS 2.0 and a second measure of symptom severity was used to estimate WHODAS’s external responsiveness to change. Spearman’s rank-order correlation values of 0.00–0.09 indicated negligible correlation, while values of 0.10–0.39 indicated weak correlation, 0.40–0.69 moderate correlation, 0.70–0.89 strong correlation and values of 0.90–1.00 very strong correlation.

## Results

We obtained sociodemographic data from most of the data-sets included in our analysis (Table 2). There were varying proportions of male and female participants across country data-sets, with the largest discrepancies found in the case of Peru and Brazil, where a notable skew towards females was observed (81.4 and 86.8%, respectively) and South Africa which was based entirely on a sample of females (100%). A large proportion of participants from the African data-sets (between 59 and 92%) reported not having completed secondary school.

**Table 2:** Sociodemographic characteristics of participants across the included data-sets

Variables	Ethiopian validation study (*N* = 150) *n* (%)	Ethiopia TaSCS trial (*N* = 324) *n* (%)	South Africa AFFIRM trial (*N* = 425) *n* (%)	India HAP trial (*N* = 493) *n* (%)	Peru CONEMO trial (*N* = 429) *n* (%)	Brazil CONEMO trial (*N* = 706) *n* (%)	Nigeria COSIMPO trial (*N* = 156) *n* (%)	Ghana COSIMPO trial (*N* = 151) *n* (%)
Study arm								
Control arm		166 (51.2)	216 (50.8)	248 (50.3)	214 (49.9)	357 (50.6)	72 (46.2)	69 (45.7)
Intervention arm		158 (48.8)	209 (49.2)	245 (49.7)	215 (50.1)	349 (49.4)	84 (53.8)	82 (54.3)
Gender								
Male	81 (54.0)	215 (66.4)	0 (0)		80 (18.7)	93 (13.2)	97 (62.2)	93 (61.6)
Female	69 (46.0)	109 (33.6)	425 (100.0)		349 (81.4)	613 (86.8)	59 (37.8)	58 (38.4)
Age								
Mean (s.d.)	30.4 (10.0)	40.5 (11.0)	27.5 (5.5)		62.9 (12.5)	55.6 (11.6)	35.9 (10.3)	30.6 (11.6)
Marital status								
Single	53 (35.3)	86 (26.5)	0 (0.0)				77 (49.4)	106 (70.2)
Married	81 (54.0)	207 (63.9)	148 (34.8)				40 (25.6)	23 (15.2)
Other (divorced, widowed or do not live together)	16 (10.7)	31 (9.7)	277 (64.2)				39 (25.0)	22 (14.6)
Highest level of education attained								
Grade 0–11	138 (92.0)	317 (96.9)	251 (59.1)				96 (61.5)	100 (66.2)
Grade 12 completed	10 (6.7)	3 (1.2)	151 (35.5)				42 (26.9)	15 (9.9)
College or above	2 (1.3)	4 (1.9)	23 (5.4)				18 (11.5)	15 (9.9)

TaSCS, Task Sharing for the Care of Severe mental disorders in a low-income country; AFFIRM, Africa Focus on Intervention Research for Mental Health; HAP, Health Activity Program; CONEMO, Emotional Control; COSIMPO, Collaborative Shared Care to Improve Psychosis.

Sociodemographic data pertaining to gender, age, marital status and education were not available for some of the data-sets.

### Factorial validity

The factor loadings of the one-factor model for the 12-item WHODAS 2.0 were within an acceptable range, exceeding 0.4, with the exception of two items from the two data-sets (see Supplementary material 1 available at https://doi.org/10.1192/bjo.2025.10778). All data-sets demonstrated model fit ranging from moderate to excellent in all of the fit indices (Table 3), with the exception of the South Africa data-set, which had a borderline RMSEA value and the Brazil data-set with an χ^2^/df value slightly higher than 3. The Ethiopian validation study data-set exhibited the best fit, with the Ethiopian TaSCS Trial data-set also performing well.

**Table 3:** Summary of model-fit indices of the one-factor model confirmatory factor analysis of the 12-item World Health Organization Disability Assessment Schedule 2.0

Fit index	Brazil	Peru	South Africa	Ethiopian TaSCS trial	Ethiopian validation study
χ^2^/d.f.	3.17	1.92	2.41	1.29	1.06
RMSEA	0.05	0.04	0.06	0.03	0.02
AIC	24871.69	14613.10	6393.95	7871.66	4866.32
BIC	25099.31	14779.52	6597.66	8064.47	5037.92
CFI	0.96	0.96	0.96	0.99	0.99
TLI	0.93	0.95	0.93	0.98	0.99
SRMR	0.04	0.04	0.037	0.029	0.02

TaSCS, Task Sharing for the Care of Severe mental disorders in a low-income country; RMSEA, root mean square error of approximation; AIC, Akaike InformationCriterion; BIC, Bayesian Information Criterion; CFI, Comparative Fit Index; TLI, Tucker–Lewis Index; SRMR, standardised root mean squared residual.

In addition to our main analysis of the baseline data-sets, we also carried out the CFA with the follow-up data-sets (see Supplementary material 2). We found that the Peru and Brazil data-sets consistently demonstrated excellent model fit, with high CFI and TLI values, and SRMR and RMSEA values well below the acceptable threshold of 0.05. In contrast, the South Africa data-set showed a marked decline in model fit from 3 months to 12 months. The Ethiopia TaSCS trial data-set exhibited a good fit both at 12 and 18 months, but the Ethiopia validation study showed a poor fit at 6 weeks’ follow-up. Factor loadings also changed as model fit indices changed across time points.

### Internal consistency of 12-item WHODAS 2.0

The internal consistency of the 12-item WHODAS 2.0 was found to be good to excellent across all settings and time points, with Cronbach’s α coefficients ranging from 0.83 to 0.97 (Table 4).

**Table 4:** Internal consistency of 12-item WHODAS 2.0 (World Health Organization Disability Assessment Schedule) across settings and time points

Data-sets	Cronbach’s α at each time point		
Ethiopia WHODAS 2.0 validation study among people with severe mental disorders (*n* = 150)	Baseline	6 weeks	
	0.95	0.97	
Ethiopia TaSCS trial data-set among people with severe mental disorders (*n* = 299)	Baseline	12 months	18 months
	0.88	0.89	0.89
South Africa AFFIRM trial on perinatal depression (*n* = 425)	Baseline – antenatal	3 months postnatal	12 months postnatal
	0.87	0.85	0.83
Peru CONEMO trial among people with depressive symptoms and hypertensive or diabetes diagnosis (*n* = 389)	Baseline	3 months	6 months
	0.83	0.87	0.87
Brazil CONEMO trial among people with depressive symptoms and hypertensive or diabetes diagnosis (*n* = 720)	Baseline	3 months	6 months
	0.83	0.87	0.87
Nigeria COSIMPO trial among people with psychoses (*n* = 156)	Baseline	3 months	6 months
	0.83	0.86	0.92
Ghana COSIMPO trial among people with psychoses (*n* = 151)	Baseline	3 months	6 months
	0.86	0.92	0.92

TaSCS, Task Sharing for the Care of Severe mental disorders in a low-income country; AFFIRM, Africa Focus on Intervention Research for Mental Health; CONEMO, Emotional Control; COSIMPO, Collaborative Shared Care to Improve Psychosis.

Cronbach’s α values are not provided for the India Health Activity Program trial as we did not obtain item-level information on WHODAS 2.0 in this data-set.

### Concurrent validity

A cross-sectional analysis was conducted for each data-set at each time point (Table 5). Moderate to strong correlations between the 12-item WHODAS 2.0 and measures of functioning or quality of life were observed, with the exception of the Global Assessment of Functioning (GAF) in Nigeria and Ghana, which showed a weak negative correlation at baseline (*r* = −0.34 and *r* = −0.28, respectively). Negative correlations between the WHODAS 2.0 and GAF were expected as higher WHODAS 2.0 scores indicate worse functioning, while higher GAF scores indicate better functioning.

**Table 5:** Correlation of 12-item WHODAS 2.0 (World Health Organization Disability Assessment Schedule) scores with scores of other functioning or quality of life measures across settings and time points (concurrent validity)

Data-sets	Comparator functioning or quality of life measure	Pearson correlation coefficients at each time point		
Ethiopia WHODAS 2.0 validation study among people with severe mental disorders (*n* = 150)	Butajira Functioning Scale	Baseline	6 weeks	
		0.88	0.92	
Ethiopia TaSCS trial among people with severe mental disorders (*n* = 299)		Baseline	12 months	18 months
		0.80	0.82	0.82
South Africa AFFIRM trial on perinatal depression (*n* = 425)	Cape Town Functional Assessment Instrument for Maternal Depression	Baseline	3 months postnatal	12 months postnatal
		0.67	0.47	0.43
Peru CONEMO trial among people with depressive symptoms and hypertensive or diabetes diagnosis (*n* = 389)	EUROQOL-5 Dimension	Baseline	3 months	6 months
		0.67	0.74	0.78
Brazil CONEMO trial among people with depressive symptoms and hypertensive or diabetes diagnosis (*n* = 720)		Baseline	3 months	6 months
		0.53	0.69	0.69
Nigeria COSIMPO trial among people with psychoses (*n* = 156)	Global Assessment of Functioning	Baseline		
		−0.34		
Ghana COSIMPO trial among people with psychoses (*n* = 151)		Baseline		
		−0.28		

TaSCS, Task Sharing for the Care of Severe mental disorders in a low-income country; AFFIRM, Africa Focus on Intervention Research for Mental Health; CONEMO, Emotional Control; COSIMPO, Collaborative Shared Care to Improve Psychosis.

In Ethiopia, the correlation between the 12-item WHODAS 2.0 scores and the Butajira Functioning Scale (BFS) scores in people with severe mental disorders was found to be strong at baseline (*r* = 0.88 and *r* = 0.80) and at follow-up (*r* = 0.92 and *r* = 0.82) in the Ethiopia WHODAS-2.0 validation study and Ethiopia TaSCS trial, respectively. In South Africa, the correlation between 12-item WHODAS 2.0 and the CFA scores among women with perinatal depression was found to be strong at baseline (*r* = 0.67) and moderate at the 3- and 12-months postnatal follow-up (*r* = 0.47 and *r* = 0.43, respectively). We found moderate to strong positive correlations between WHODAS 2.0 scores and EQ-5D scores in both Brazil and Peru in assessments conducted at baseline and follow-up periods (*r* = 0.53–0.78).

### Convergent validity

At baseline, we found weak to moderate correlation between 12-item WHODAS 2.0 scores and BPRS scores (*r* = 0.29 and *r* = 0.41 in the Ethiopia validation study and the Ethiopia TaSCS trial, respectively), PHQ-9 scores (*r* = 0.30 and *r* = 0.42 in Peru and Brazil, respectively), EPDS scores (*r* = 0.36 in South Africa), HDRS scores (*r* = 0.37 in South Africa), and PANSS scores (*r* = 0.24 in Nigeria and *r* = 0.34 in Ghana). There was an increase in correlation coefficients across settings at first follow-up, with the exception of the correlation with the HDRS measure in South Africa) (Table 6).

**Table 6:** Correlation of 12-item WHODAS 2.0 (World Health Organization Disability Assessment Schedule) scores with scores of symptom measures across settings and time points (convergent validity)

Data-sets	Comparator symptom measure	Pearson correlation coefficients at each time point		
Ethiopia WHODAS 2.0 validation study among people with severe mental disorders (*n* = 150)	Brief Psychiatric Rating Scale – Expanded	Baseline	6 weeks	
		0.29	0.47	
Ethiopia TaSCS trial among people with severe mental disorders (*n* = 299)		Baseline	12 months	18 months
		0.41	0.58	0.41
South Africa AFFIRM trial on perinatal depression (*n* = 425)	Edinburgh Postnatal Depression Scale	Baseline	3 months postnatal	12 months postnatal
		0.36	0.40	0.22
	Hamilton Depression Rating Scale	Baseline	3 months postnatal	12 months postnatal
		0.37	0.35	0.19
India HAP trial among people with moderately severe to severe depression in primary health care (*n* = 437)	Patient Health Questionnaire-9	Baseline	3 months	12 months
			0.70	0.67
Peru CONEMO trial among people with depressive symptoms and hypertensive or diabetes diagnosis (*n* = 389)		Baseline	3 months	6 months
		0.30	0.74	0.69
Brazil CONEMO trial among people with depressive symptoms and hypertensive or diabetes diagnosis (*n* = 720)		Baseline	3 months	6 months
		0.42	0.65	0.63
Nigeria COSIMPO trial among people with psychoses (*n* = 156)	Positive and Negative Syndrome Scale	Baseline	3 months	6 months
		0.24	0.59	0.45
Ghana COSIMPO trial among people with psychoses (*n* = 151)		Baseline	3 months	6 months
		0.34	0.68	0.82

TaSCS, Task Sharing for the Care of Severe mental disorders in a low-income country; AFFIRM, Africa Focus on Intervention Research for Mental Health; HAP, Health Activity Program; CONEMO, Emotional Control; COSIMPO, Collaborative Shared Care to Improve Psychosis.

Moderate correlation was observed between the 12-item WHODAS-2.0 and BPRS scores at follow-up in Ethiopia (*r* = 0.41–0.58). A moderate to strong correlation was found between the 12-item WHODAS 2.0 and PANSS scores at both the 3- and 6-month follow-ups in Nigeria (*r* = 0.59 and *r* = 0.45), and in Ghana (*r* = 0.68 and *r* = 0.82). At the 3-month follow-up, there was a moderate to strong correlation between WHODAS 2.0 and PHQ-9 scores in India, Peru and Brazil (*r* = 0.70, *r* = 0.74, and *r* = 0.65, respectively). The moderate correlation between the WHODAS 2.0 and PHQ-9 scores persisted at the 6-month follow-up in Peru and Brazil (*r* = 0.69 and 0.63, respectively) and the 12-month follow-up in India (*r* = 0.67). This was contrary to what was observed in South Africa where correlations between the 12-item WHODAS 2.0 and EPDS scores declined between the 3- and 12-month postnatal follow-up (*r* = 0.40 and *r* = 0.22, respectively). We observed the same pattern with respect to correlations between the WHODAS 2.0 and Hamilton Depression Rating Scale (HDRS) scores in the 3- and 12-month follow-ups (*r* = 0.35 and *r* = 0.19, respectively).

### Responsiveness to change

There were significant changes in the 12-item WHODAS 2.0 scores from baseline to the 3-month follow up in Brazil, Peru, South Africa, Nigeria and Ghana (*p* < 0.01) (Table 7). A significant change in WHODAS 2.0 scores was also observed in Brazil, Peru, Nigeria and Ghana (*p* < 0.01) from baseline to the 6-month follow-up. Similarly, there was a significant change in the WHODAS 2.0 scores from baseline to the 12-month postnatal follow up in South Africa (*p* < 0.01). In contrast, there was no significant change in the 12-item WHODAS 2.0 scores from baseline to the 6-week follow-up in the Ethiopian validation study (*p* > 0.05). Overall, the internal sensitivity to change of WHODAS-2.0 was found to be large in most study settings (Cohen’s *d*: 0.67–1.94), with the exception of the Brazil, Ethiopia validation and TaSCS trial where small- and medium-effect sizes were observed (Cohen’s *d* = 0.28, 0.12 and 0.06, respectively).

**Table 7:** Internal responsiveness to change of the 12-item World Health Organization Disability Assessment Schedule 2.0 across settings and time points

Data-set^a^	Mean score (s.d.) at each time point			Paired *t*-test between baseline and time 1 (*p*-value)	Paired *t*-test between baseline and time 2 (*p*-value)	Effect size between baseline and time 1	Effect size between baseline and time 2
Ethiopia/validation study	Baseline	6 weeks					
	31.54 (13.10)	30.02 (13.90)		0.82 (0.21)		0.12	
Ethiopia/TaSCS trial	Baseline	12 months	18 months				
	10.02 (6.72)	9.65 (7.19)	9.04 (7.01)	0.88 (0.19)	2.35 (<0.02*)	0.06	0.15
South Africa/AFFIRM	Baseline	3 months postnatal	12 months postnatal				
	15.53 (8.68)	7.72 (9.10)	7.17 (5.31)	9.40 (<0.01**)	9.25 (<0.01**)	0.90^††^	0.96^††^
Peru/CONEMO	Baseline	3 months	6 months				
	19.18 (8.19)	13.65 (8.10)	12.85 (7.49)	8.46 (<0.01**)	9.43 (<0.01**)	0.67^††^	0.76^††^
Brazil/CONEMO	Baseline	3 months	6 months				
	14.83 (8.95)	12.37 (9.08)	11.67 (9.25)	5.82 (<0.01**)	6.98 (<0.01**)	0.28^†^	0.35^†^
Nigeria/COSIMPO	Baseline	3 months	6 months				
	28.38 (10.04)	19.20 (6.78)	14.45 (4.69)	7.37 (<0.01**)	11.03 (<0.01**)	0.91^††^	1.41^††^
Ghana/COSIMPO	Baseline	3 months	6 months				
	31.13 (10.16)	12.35 (6.23)	13.80 (4.07)	5.26 (<0.01**)	7.81 (<0.01**)	1.21^††^	1.94^††^
Nigeria and Ghana/COSIMPO	Baseline	3 months	6 months				
	29.57 (10.07)	19.09 (6.62)	14.45 (4.57)	9.01 (<0.01**)	13.01 (<0.01**)	1.06^††^	1.47^††^

TaSCS, Task Sharing for the Care of Severe mental disorders in a low-income country; AFFIRM, Africa Focus on Intervention Research for Mental Health; CONEMO, Emotional Control; COSIMPO, Collaborative Shared Care to Improve Psychosis.

a Analysis of the Indian Health Activity Program trial was not included in this table as this dataset did not include baseline data (i.e. data collected before the intervention was administered). Pooled estimates for the Nigeria and Ghana samples from the COSIMPO trial are also presented in this table due to the small sample size in these datasets.

**p < 0.01 and *p < 0.05; ^††^large effect size according to Cohen’s *d* and ^†^medium effect size according to Cohen’s *d*.

Changes in the WHODAS 2.0 scores were significantly associated with changes in the PHQ-9 scores in Peru at both the 3- and 6-month follow-up (rho = 0.35 and 0.37 respectively, *p* < 0.01), whereas a significant association was observed only at the 6-month follow-up period in Brazil (rho = 0.53, *p* < 0.01) (Table 8). In the TaSCS trial in Ethiopia, changes in the WHODAS 2.0 scores were significantly associated with changes in the BPRS scores at the 12-month follow-up (rho = 0.17, *p* < 0.05) but not at the 18-month follow-up (*p* > 0.05). In the Ethiopia validation study, changes in the WHODAS 2.0 scores were significantly associated with change in the BPRS scores (rho = 0.53, *p* < 0.01).

**Table 8:** External sensitivity to change of the 12-item World Health Organization Disability Assessment Schedule 2.0 compared with mental health symptom measures across settings and time points

Setting/data-set	Comparator symptom measure	Spearman’s rank difference correlation coefficient from baseline to time 1: ρ (*p*-value)	Spearman’s rank difference correlation coefficient from baseline to time 2: ρ (*p*-value)
Ethiopia validation study	Brief Psychiatric Rating Scale – Expanded	6 weeks	
		0.53 (<0.01**)	
Ethiopia/TaSCS trial		12 months	18 months
		0.17 (<0.05*)	0.11 (0.16)
South Africa/AFFIRM	Edinburgh Postnatal Depression Scale	3 months postnatal	12 months postnatal
		0.24 (<0.05*)	0.14 (0.13)
Peru/CONEMO	Patient Health Questionnaire-9	3 months	6 months
		0.35 (<0.01**)	0.37 (<0.01**)
Brazil/CONEMO		3 months	6 months
		0.19 (0.20)	0.53 (<0.01**)
Nigeria/COSIMPO	Positive and Negative Syndrome Scale	3 months	6 months
		0.43 (<0.01**)	0.30 (<0.02*)
Ghana/COSIMPO		3 months	6 months
		0.31 (0.15)	0.40 (0.13)
Nigeria and Ghana/COSIMPO		3 months	6 months
		0.47 (<0.01**)	0.28 (<0.01**)

TaSCS, Task Sharing for the Care of Severe mental disorders in a low-income country; AFFIRM, Africa Focus on Intervention Research for Mental Health; CONEMO, Emotional Control; COSIMPO, Collaborative Shared Care to Improve Psychosis.

Analysis of the Indian Health Activity Program trial was not included in this table as this data-set did not include baseline data (i.e. data collected before the intervention was administered). Pooled estimates for the Nigeria and Ghana samples are also presented in this table due to the small sample size in these data-sets.

**p < 0.01 and *p < 0.05.

In South Africa, changes in the WHODAS 2.0 scores were significantly correlated with changes in the EPDS scores at the 3-month postnatal follow-up period although the correlation was weak (rho = 0.24, *p* < 0.05); this correlation dissipated at the 12-month postnatal follow-up (*p* = 0.13). In Nigeria, a change in the WHODAS 2.0 scores was moderately and significantly correlated with a change in the PANSS scores at the 3-month follow-up (rho = 0.43, *p* < 0.01), whereas a weak yet significant correlation was observed at the 6-month follow-up period (rho = 0.30, *p* < 0.02). Overall, the external sensitivity to change of WHODAS 2.0 was found to be weak to moderate across different study settings and time points.

## Discussion

This study assessed the psychometric properties of the 12-item version of the WHODAS 2.0 across diverse settings and among individuals with various mental health conditions using secondary data from seven LMICs. Based on the existing evidence, we hypothesised that the 12-item WHODAS 2.0 would demonstrate a single-factor structure, good internal consistency, moderate correlation with measures of symptom severity (convergent validity), moderate correlation with measures of functioning or quality of life (concurrent validity), significant improvements in WHODAS 2.0 scores over time (internal sensitivity to change), and moderate correlation between changes in WHODAS 2.0 scores and changes in scores of measures of symptom severity (external sensitivity to change).

Several previous cross-sectional studies across general populations, or groups with physical or mental health disorders show that a one-factor structure fits well with the 12-item WHODAS 2.0.^6^ Our analysis of LMIC data-sets shows that the one-factor structure of the 12-item WHODAS 2.0 has an acceptable fit at baseline in mental health populations, but is not stable across time in all settings and cultures. While the Peru, Brazil and the Ethiopia TaSCS trial data-sets consistently demonstrated excellent model fit across follow-up time points, the South Africa and the Ethiopia validation study data-sets showed a marked decline in model fit from baseline to follow-up. One possible reason is that interventions are expected to cause changes in functioning, but these changes may not be the same across all aspects of daily activities and tasks, which will likely reduce the correlation among the items in the scale. Factor loadings in all data-sets changed as model-fit indices changed across time points. These findings emphasise the importance of assessing model fit not only across study settings but also over time in future studies.

In line with our hypotheses, we found a consistently high internal consistency of the 12-item version of WHODAS 2.0 across settings and time points. Our study findings align with the existing evidence base, which has demonstrated a good to excellent internal consistency of the WHODAS 2.0 in studies conducted across mental and physical health conditions.^9,15,38^

While we hypothesised that the 12-item WHODAS 2.0 would be moderately correlated with measures of symptom severity in populations with mental health conditions, our study findings demonstrated weak to moderate correlation at baseline, which generally increased to moderate or strong associations in subsequent follow-ups. At follow-up, both symptoms and functioning are likely to improve with higher variability among participants, thus resulting in an increase in the correlation between scores on symptom and functioning measures.^8^ While our findings mirror the weak correlation identified in a previous study in another LMIC (India),^21^ they did not align with prior evidence stemming from high-income countries showing moderate to strong convergent validity between the WHODAS 2.0 and symptom measures such as the PHQ-9 and Kessler 6 in mental health populations.^9,11,15^ Weak to moderate correlations between the WHODAS 2.0 and measures of symptom severity at baseline may be due to an underestimation of functional impairment when self-reported by individuals with a mental health condition, which tends to improve as the individual’s condition improves.^20^ Additionally, aside from symptom severity, external factors such as stigma and poverty may also influence a person’s levels of functional impairment.^8^ Differences in social and economic conditions across study settings may also explain the variability of results observed in the current study.

Consistent with our hypotheses, moderate to strong correlations between WHODAS 2.0 scores and the scores of other functioning or quality of life measures were observed across most study settings and time points, with the exception of the GAF in Ghana and Nigeria where a weak correlation with the GAF was observed. This may be due to differences in the nature of these measures. While GAF is a single-item clinician-rated measure of functioning, WHODAS 2.0 is a multi-item and multi-domain self- or interviewer-administered measure of functional impairment. The weak correlation observed within our study may be indicative of differences in the perceptions of functional impairment due to psychosis between clinicians and patients. Previous studies have demonstrated that particularly among people with psychosis, patient- and clinician-reported outcomes highlight differing aspects of the patient’s functioning or symptoms.^39,40^ Additionally, GAF has been criticised by experts in the field for its inconsistent representation of functional impairment, which may contribute to its weak correlation with the WHODAS 2.0.^41^ Setting may also be playing a role in the differences seen, as our findings contrast with studies from high-income settings, which found moderate correlations between WHODAS 2.0 and the GAF among populations with a range of mental disorders in Spain and among populations with schizophrenia and schizoaffective disorders in Japan.^14,15^

Overall, our study findings largely align with previous evidence stemming from high-income countries indicating that WHODAS 2.0 has moderate to strong concurrent validity across various health conditions.^9,11,38^ Collectively, these findings support the CMMH’s recommendations for the routine use of WHODAS 2.0 for the assessment of functioning across a range of mental health conditions and settings.^7,42^

Consistent with our hypothesis, the 12-item WHODAS 2.0 was able to pick up on changes as a result of treatment interventions with large-effect sizes observed across most study settings, indicating internal responsiveness to change. However, contrary to our hypothesis, external responsiveness to change exhibited variability, with weak to moderate correlations between changes in WHODAS 2.0 and symptom scores across study settings and time points. These findings underscore the nuanced and varying patterns of external sensitivity to change across different settings and time points. Specifically, the variability of the results observed in relation to external responsiveness to change may be partially attributable to differences in the length of follow-up periods across the included studies. Our study findings may also be attributed to a recognised clinical phenomenon wherein alterations in functioning typically trail behind changes in symptoms.^43^ Consequently, the correlation between changes in WHODAS 2.0 scores and symptom changes may sometimes be moderate or weak.^44^ The weak or moderate correlation between changes in symptom severity and disability scores may also be explained by additional factors that influence these scores, such as stigma and the personal and socioeconomic circumstances of participants.^45^

The acceptable psychometric properties of WHODAS 2.0 demonstrated in our study findings suggest its importance as a reliable and valid tool for disability assessment in cross-cultural settings and across different mental health conditions. Our findings are also in line with the previously reported Ethiopian validation study that showed that the psychometric properties of WHODAS 2.0 were comparable to a newly developed, contextually-tailored measure of functioning (the BFS).^8^ This does not detract from the value of locally developed measures, which capture specific tasks of salience to that particular population and may be especially useful in clinical settings, but our findings do indicate that WHODAS 2.0 has advantages for comparative epidemiological studies in LMICs, as was found for dementia.^46^ The widespread use of WHODAS 2.0, likely to continue and even increase as a result of the CMMH initiative for global health funders,^7,42^ will be important for informing global burden of disease estimates (currently LMIC data is under-represented).^3^

Overall, this study adds to the existing evidence base by providing a novel assessment of the cross-cultural performance of the WHODAS 2.0 as a measure of functioning across seven LMICs. Given that current population-based assessments of mental health conditions, particularly depression, often focus solely on symptom criteria, broadening the criteria to include associated functional impairments could enhance the accuracy of prevalence estimates.^47^ It is worth noting that research involving people with lived experience on the usefulness and interpretability of the WHODAS 2.0 is scarce. We identified only one study that collected qualitative information on the interpretability of the WHODAS 2.0 in their local setting.^8^ Moving forward, future work involving perspectives from people with lived experience on adaptations to the WHODAS 2.0 can offer important insights that enhance the utility of this measure across different settings.

The strengths of the current study lie in the assessment of the psychometric properties of WHODAS 2.0 across a diverse range of LMICs and among individuals with various mental health conditions. Assessing both internal and external sensitivity to change is a robust indicator of validity, albeit not commonly implemented due to the need for follow-up data. Therefore, being able to evaluate the responsiveness to change of the WHODAS 2.0 among seven LMICs is a key strength to this study that addresses an important research gap.

In terms of the study limitations, the sample size was small for some of the analyses conducted, particularly of external responsiveness to change. However, overall, the results were consistent across different settings. Furthermore, this study relied on data that had already been collected which has inherent limitations, particularly regarding the completeness of variables and data. Data-sets included within this study stemmed from populations of differing gender, age and mental health conditions and were conducted at varying time points. This can impact the generalisability of the results. Furthermore, one of the included studies used the 36-item WHODAS 2.0, but we relied on the extraction of data from individual items pertaining to the 12-item WHODAS 2.0. Differences in administration time for the 36-item WHODAS 2.0 may have influenced completion rates and findings. Lastly, we recognise that WHODAS 2.0, a disability measure, can be seen as reinforcing a focus on deficits. Calls for capability-focused measures have been made and it will be important to also establish the cross-cultural validity of such measures.

In conclusion, the 12-item WHODAS 2.0 consistently demonstrated acceptable fit to a one-factor model, internal consistency, concurrent validity and internal sensitivity to change in diverse settings and in people with diverse mental health conditions. In contrast, high levels of variability were observed in relation to WHODAS 2.0’s convergent validity and external responsiveness to change across countries and time points that warrant further investigation. Our findings suggest that the WHODAS 2.0 is a suitable measure for the comparative assessment of functioning among people with mental health conditions within LMIC settings, and support its utility as a key outcome measure.

The evidence to date focuses predominantly on evaluating the psychometric properties of WHODAS 2.0 within the context of high-income settings. In light of this, our study provides novel insights in relation to its performance in LMICs. However, additional work is still needed to examine the content validity, and internal and external responsiveness to change of the WHODAS 2.0 in other LMIC settings, as well as other psychometric properties such as measurement invariance. Involving people with lived experience to study the content validity and interpretability of items and scores of the WHODAS 2.0 will also be useful. Further evaluation of the WHODAS 2.0 in LMICs will enable the development of a more robust and well-balanced evidence base, and support adaptations of the WHODAS 2.0 for different contexts and study settings.

## Supporting information

Mihretu et al. supplementary material Mihretu et al. supplementary material

## Data Availability

This study used secondary data made available to us from respective principal investigators upon request and hence the authors do not have permission to share. Requests to access the data should be directed to corresponding authors of the respective publications of the original data.

## Supplementary material

The supplementary material is available online at https://doi.org/10.1192/bjo.2025.10778
