# Supplementary material for: Evaluating the reliability and validity of the 12-item WHODAS 2.0 among people with mental health conditions in seven low- and middle-income countries: analysis of secondary data
Source: BJPsych Open. 2025 Oct 1;11(6):e231. doi: 10.1192/bjo.2025.10778 (PMC12529340; doi:10.1192/bjo.2025.10778)
Supplement: Mihretu et al. supplementary material [file S2056472425107783sup001.docx]

Supplementary Material 1

Table S1: Factor loadings per WHODAS item per dataset

| Dataset | Official WHODAS item name | Respective WHODAS item as it the dataset | Factor loading |
| --- | --- | --- | --- |
| CONEMO Peru | Standing for long | Pdas1 | .54 |
|  | Taking care of household responsibilities | Pdas2 | .66 |
|  | Learning a new task | Pdas3 | .49 |
|  | Joining in community activities | Pdas4 | .51 |
|  | Emotionally affected | Pdas5 | .58 |
|  | Concentrating | Pdas6 | .49 |
|  | Walking a long distance | Pdas7 | .63 |
|  | Washing | Pdas8 | .50 |
|  | Dressing | Pdas9 | .57 |
|  | Dealing with people | Pdas10 | .32 |
|  | Maintaining a friendship | Pdas11 | .33 |
|  | Day-to-day work/school | Pdas12 | .68 |
| CONEMO Brazil | Standing for long | Pdas1 | .60 |
|  | Taking care of household responsibilities | Pdas2 | .68 |
|  | Learning a new task | Pdas3 | .48 |
|  | Joining in community activities | Pdas4 | .55 |
|  | Emotionally affected | Pdas5 | .54 |
|  | Concentrating | Pdas6 | .51 |
|  | Walking a long distance | Pdas7 | .61 |
|  | Washing | Pdas8 | .51 |
|  | Dressing | Pdas9 | .54 |
|  | Dealing with people | Pdas10 | .33 |
|  | Maintaining a friendship | Pdas11 | .34 |
|  | Day-to-day work/school | Pdas12 | .71 |
| AFFIRM South Africa | Standing for long | standc_1 | .75 |
|  | Taking care of household responsibilities | respc_1 | .71 |
|  | Learning a new task | learnc_1 | .73 |
|  | Joining in community activities | actc_1 | .58 |
|  | Emotionally affected | affectc_1 | .59 |
|  | Concentrating | concc_1 | .66 |
|  | Walking a long distance | walkc_1 | .71 |
|  | Washing | washc_1 | .55 |
|  | Dressing | dressc_1 | .54 |
|  | Dealing with people | dealc_1 | .53 |
|  | Maintaining a friendship | friendc_1 | .46 |
|  | Day-to-day work/school | workc_1 | .73 |
| AFFIRM Ethiopian TaSCS trial | Standing for long | whodas01 | .52 |
|  | Taking care of household responsibilities | whodas02 | .60 |
|  | Learning a new task | whodas03 | .77 |
|  | Joining in community activities | whodas04 | .66 |
|  | Emotionally affected | whodas05 | .67 |
|  | Concentrating | whodas06 | .66 |
|  | Walking a long distance | whodas07 | .70 |
|  | Washing | whodas08 | .62 |
|  | Dressing | Whodas9 | .53 |
|  | Dealing with people | whodas10 | .40 |
|  | Maintaining a friendship | whodas11 | .65 |
|  | Day-to-day work/school | whodas12 | .66 |
| AFFIRM Ethiopia validation study | Learning a new task | LEARNTAS | .88 |
|  | Standing for long | STANDING | .73 |
|  | Washing | WASHING | .67 |
|  | Dressing | DERESSED | .66 |
|  | Dealing with people | DEALPEOP | .77 |
|  | Maintaining a friendship | MAIFRIEN | .73 |
|  | Day-to-day work/school | WORKSCHO | .84 |
|  | Joining in community activities | COMMUACT | .85 |
|  | Emotionally affected | EMOTION | .75 |
|  | Concentrating | CONCENTR | .76 |
|  | Taking care of household responsibilities | HOUSRESP | .85 |
|  | Walking a long distance | WALKLONG | .65 |

Figure S1: One factor model for the CONEMO Peru baseline dataset

Figure S2: One factor model for the CONEMO Brazil baseline dataset

Figure S3: One factor model for the AFFIRM South Africa baseline dataset

Figure S4: One factor model for the AFFIRM Ethiopia TaSCS trial baseline dataset

.

Figure S5: One factor model for the AFFIRM Ethiopia validation study baseline dataset

**Supplementary Material 2:**

Table S2: Summary of one factor model fit indices across datasets and time points

| **Datasets** | **Time points** | **χ²/df** | **RMSEA** | **AIC** | **BIC** | **CFI** | **TLI** | **SRMR** |
| --- | --- | --- | --- | --- | --- | --- | --- | --- |
| AFFIRM South Africa | 3 month | 1.72 | 0.054 | 5944.470 | 6085.647 | 0.949 | 0.932 | 0.050 |
|  | 12 month | 7.15 | 0.139 | 6722.40 | 6907.05 | 0.875 | 0.800 | 0.093 |
| AFFIRM Ethiopian TaSCS trial | 12 month | 4.63 | 0.09 | 7379.210 | 7513.959 | 0.920 | 0.902 | 0.060 |
|  | 18 month | 3.35 | 0.031 | 6654.90 | 6825.12 | 0.943 | 0.914 | 0.047 |
| AFFIRM Ethiopia validation study | 6 week | 6.43 | 0.191 | 5123.263 | 5240.6 | 0.820 | 0.767 | 0.070 |
| CONEMO Peru | 3 month | 1.63 | 0.039 | 13476.4 | 13649.4 | 0.984 | 0.978 | 0.037 |
|  | 6 month | 2.52 | 0.062 | 15033.7 | 15204.5 | 0.962 | 0.946 | 0.045 |
| CONEMO Brazil | 3 month | 1.75 | 0.031 | 46143.2 | 46353.0 | 0.985 | 0.978 | 0.026 |
|  | 6 month | 1.03 | 0.007 | 60137.086 | 60363.534 | 0.999 | 0.999 | 0.022 |

***See main manuscript Table 3 for the model fit indices of the baseline datasets*

*Figure S6: One factor model for the AFFIRM South African dataset at 3 months*

*Figure S7: One factor model for the AFFIRM South African dataset at 12 months*

*Figure S8: One factor model for the AFFIRM Ethiopian validation study dataset at 6 weeks*

*Figure S9: One factor model for the AFFIRM Ethiopian TaSCS trial dataset at 12 months*

*Figure S10: One factor model for the AFFIRM Ethiopian TaSCS trial datasets at 18 months*


*Figure S11: One factor model for the CONEMO Peru dataset at 3 months*

*Figure S12: One factor model for the CONEMO Peru dataset at 6 months*
*Figure S13: One factor model for the CONEMO Brazil dataset at 3 months*

*Figure S14: One factor model for the CONEMO Brazil dataset at 6 months*
